# Round the Hospitals

**Published:** 1918-02-23

**Authors:** 


### ROUND THE HOSPITALS.

It is with pleasure we announce that His Majesty the King has sent a donation of £100 to the Nation's Fund for Nurses. Governors of hospitals, and especially those actively engaged in their management, by contributing to this Fund without delay, would exhibit their patriotism and demonstrate their appreciation of the personal service which the British Women's Hospital Committee, 21 Old Bond Street, London, W., are zealously rendering to the best interests of the nursing profession, with such excellent results.

☛ Previous articles appeared January 19, p. 337; January 26, p. 357; and February 9, p. 401.

**ROUND THE HOSPITALS—(*continued*).**

The resignation of Miss Ray, R.R.C., sister-matron of King's College Hospital, makes a notable gap in the nursing world. During the twelve years of her term of office, Miss Ray has steered her department through vicissitudes of no common order. During the years which witnessed the gradual approach towards completion of the new hospital at Denmark Hill, labours of a most exacting description fell upon those in authority. The plans for the nursing home, the kitchens, store-rooms, ward kitchens, bath-rooms, sinks, etc., were all worked out by the architects in full consultation with the sister-matron. Studied attentively, they represent a store-house of invention, and it is no secret that Miss Ray's advice and suggestions were closely followed and had no small share in making the new King's a model among modern institutions in regard to nursing and domestic premises. The strain of work involved by the working out of the stupendous mass of detail might have taxed the powers of a woman of full leisure, and it must be remembered that throughout this entire time Miss Ray was keeping the training-school at the old hospital up to its former reputation, and inspiring those who helped her to uphold its honour, with infinite patience and determination. During the slow months which saw the gradual transfer of patients and staff to the new premises, nothing but the most carefully calculated plans, thought out in every minute particular, could have preserved the double establishments from confusion and dismay. But the brilliant success which marked each stage of the transfer was in some respects less remarkable than the indomitable spirit which kept things together during that waiting period, when nothing in the old hospital could be renewed, when by sure degrees shabbiness settled down upon every part of the building, and every makeshift had to be employed in order to carry on without letting anyone suffer actual hardship. Those who worked in concert with Miss Ray through those times received a lesson in dominating circumstances they will cherish to their lives' end.

No sooner was all in harmonious working order, before, indeed, the entire premises were in use, than war broke out. From its position as 4th London General Hospital, King's College Hospital came at once under military rule. A large proportion of the wards were given up to sick and wounded soldiers, and a thorough reorganisation became necessary. At the same time many practically irreplaceable members of the nursing staff were called up and the training-school was once more subjected to a severe test. Through it all, Miss Ray preserved the quiet spirit of concentration on essential things which distinguishes women of marked administrative ability. To be in her presence, even to watch her pass along the corridors in her scarlet cape, with the air of gentle dignity which calmed down all fuss and worry in those who approached her, was to be penetrated with a sense that the talent for co-ordinating detail and descending to the root of practical matters is the outcome of a mystic quality which cannot rest satisfied with anything short of perfection. Certainly there are to-day many examples of the combination of the deeply spiritual and the intensely practical among the women who have raised the status of British nurses to their present high level.

It is of the first importance that the financial administration of the College of Nursing should rest upon the soundest business basis. The Bolos, following their uninterrupted career of misrepresentation, have tried to mislead and disturb the confidence of trained nurses in the College by repeating a statement to the effect that the whole of the registration fees paid by the nurses to the College have so far been more than absorbed by the expenses incurred. This statement has no foundation in fact, as will be demonstrated, we are glad to know, in the audited accounts which will be published in the annual report due in March next. We have further the satisfaction to make the important announcement that the Hon. Sir Wm. H. Goschen, K.B.E., the eminent financier, and Miss Sidney Browne, R.R.C., have been appointed co-honorary treasurers, with Mr. Comyns Berkeley, M.C. (Cantab.), of the College of Nursing. This announcement will gratify trained nurses among The Hospital and *Nursing Mirror* readers, who have already registered with the College on the Editor's invitation, for it fulfils the guarantee the Editor gave them when inviting their co-operation. Unity is now admitted to be essential to the speedy fulfilment of the aims and ambitions of trained nurses who love their profession, and with their friends are uniting solidly to promote these ends. If the 8,000 nurses who have now registered with the College of Nursing are increased to 10,000 by March 31 through the co-operation of readers of The Hospital and the *Nursing Mirror*, it is certain that the number will grow to 20,000 on the College Register within the next twelve months, when victory and independence will be theirs. The great majority of trained nurses, being representative members of the nursing profession, share the view held by the independent speakers at the R.B.N.A. meeting in Liverpool, that those who are unprejudiced and wise will combine and secure unity in nursing affairs. The Liverpool meeting and its teachings have been appreciated throughout the nursing profession, and unity may be regarded as certain, for further waste of time on lost causes and red herrings will not be tolerated so far as British nursing is concerned.

Our readers will note that the Medical Honorary Secretary of the Royal British Nurses' Association, despite his high qualifications, has not, so far, made the *amende honorable* in regard to the proved misstatements in his speech at Liverpool, which the two professions took for granted he would have spontaneously and promptly published.

The proceedings which took place at Liverpool on February 8 at the Royal British Nurses' Association meeting, which were fully reported in our

**ROUND THE HOSPITALS—*(continued)*.**

issue of February 16, pages 424-7, were marked by an acrimony which filled the Chairman and the audience alike with amazement. Those present were prepared for a statement from Mr. Paterson and from Miss Macdonald of the causes which led the Association to decline the Supplemental Charter offered by the Privy Council and break off negotiations with the College of Nursing. But the bitter invectives launched by Mr. Paterson, not merely against the College but against the governing bodies of voluntary hospitals in general, were certainly neither expected nor approved. When the speaker, after declaiming against the principle under which administrators are appointed to govern the great voluntary institutions of the country, went on to remark that "for the chairman of a large hospital probably a crossing-sweeper was good enough" he lost once and for all the attention of his hearers, and for all the effect that he made, after this, was merely talking to the winds.

It is pretty clear that the attempt to represent "many thousands of nurses" as resentful of the efforts made by the British Women's Hospital Committee is petering out. The Helena Benevolent Fund was certainly not got together out of nurses' earnings, but was raised, so far as the major part of it is concerned, at a bazaar. All Benevolent Funds have their origin in an appeal to public generosity. The whole voluntary principle in this country rests on the indisputable fact that as a nation the British prefer to do these things for themselves, and give out of their abundance rather than raise the money by taxation.

Do any think that the benefits conferred by the State or by municipalities out of public moneys are more delicately conferred or more grateful to the recipients than those which are freely raised by public subscription and formed into public endowments? If so, they must be singularly ill-informed in the history of rate- and State-supported hospitals and other institutions. The spirit of humanity which inspires the free gift illuminates its whole course from the pocket of the giver to its ultimate destination as an engine for redressing hardship, promoting education, or solidifying endowed institutions. Miss Gibson was on absolutely sure ground when she represented the gift to nurses, now gathering like a snowball, as a debt due from the nation to the nurses. The public is not to be restrained from paying this most just debt.

But we venture to ask, in this connection, what nurses are resentful at the idea of a national offering to nurses? Is it those who are worn out with their long toil, have lost brilliant hopes, and have nothing but a tiny pittance to look forward to for the rest of their lives? We think not. But if it be some who have continued their earning career throughout the war unaffected by its terrible demands on the vital forces, then we would ask, "By what right do you speak for your sisters? You have not shared their labours or their sorrows, you cannot bring them help in their necessities. All that is required of you is to stand aside and see that you do not hinder a work of mercy in which it would almost seem you are not particularly interested."

The Rev. A. Lombardini, chaplain of the Church of St. Elizabeth, Kensington Infirmary, Marloes Rd., W., has taken the lead in a matter of world-wide importance, the rendering of a public tribute to the nurses who have given their lives for their country. The Dean and Chapter of St. Paul's, with instant perception of national feeling, have expressed their desire that the proposed commemoration shall take the form of a service in St. Paul's Cathedral, and it is hoped that a date may be fixed two or three weeks after Easter. Mr. Lombardini is compiling a Roll of Honour, and will be grateful to any matrons or sisters who will supply him with the names of nurses who have died at their post during the war or in consequence of injuries or sickness arising out of their nursing duties, with particulars.

Addenbrooke's Hospital at Cambridge has taken timely note of the increased value of the nurse's work at the present, and has made a handsome revision of salaries accordingly. Under the new scale probationers receive during their four years respectively £12, £16, £24, and £30 a year, a rate which should ensure that the supply of suitable candidates does not run short, though Addenbrooke's has always enjoyed, to a special degree, a good choice of local women anxious to be trained in their own hospital. Ward sisters receive under the new arrangement £40, £45, and £50 in their first, second, and third years respectively. Other appointments open are: *x*-ray sister, £45, £50, £55; night sister, £45, £50; assistant matron, £60, £65, £70. The result of the new scale is to give every inducement to women of administrative ability to remain in the service of the hospital after they have received their certificate, and to lessen the competition of the private nurses' institutes, which are apt to lure some of the best nurses away from their training-schools the moment their training is complete.

The committee of the Royal Southern Hospital, Liverpool, has recently revised the scale of nursing salaries, and has adopted the following increases from January 1 in the current year. Probationers, who formerly received £8, £15, £20, are now to receive £12, £18, £24. The out-patient sister, theatre and ward sister receive £40, £42, £45, with £5 bonus, in lieu of £40 and bonus. In the case of the sister of Victoria Ward (at present military) the bonus is doubled. The night sister, who formerly received £40, with a bonus of £5, is now to have £45, £47, £50, with the same bonus. The housekeeping sister rises from £50 to £60; as does also the assistant matron, who receives in addition a bonus of £10. The nurses on the private staff receive an

**ROUND THE HOSPITALS—(*continued*).**

increase of £5 in their first and second year's service. The total cost of these alterations amounts to £334 a year.

---

The Royal Southern Hospital has, moreover, recently inaugurated an admirable Nurses' Relief Fund which already amounts to nearly £3,000, the interest of which is to be used for the purpose of sending sick nurses, should they require such aid, to the seaside or country to recuperate. Should this fund grow as is anticipated, the intention is to extend its scope so that the training fees may be paid for any of the nursing staff who may wish to sit for the massage and maternity certificates. All this speaks a thoroughly progressive spirit, and should prove of immediate value in stimulating the training-school. We are quite sure that the authorities have only to formulate plans, for the better payment and advantage of their nurses, to find the money coming rapidly in to carry them out.

---

The revised list, for the current year, of institutions, teachers, and examiners attached to the Central Midwives Board for Scotland shows that midwifery training is still concentrated in very few hands in the Northern Kingdom. Edinburgh has five institutions giving recognised midwifery training; Glasgow, one; Dundee, one; Aberdeen, one; Govan, one. In addition, one Poor-Law Institution, and one only, the Glasgow Eastern District Hospital, is recognised. Five certified midwives in Edinburgh are approved for signing certificates under Rule C, one in Glasgow, one in Paisley, one in Govan. Sir J. Halliday Croom has been re-elected chairman of the Board, and Dr. J. Haig Ferguson deputy-chairman.

---

American matrons are becoming as keen about economy as their English *confrères*. Miss Mary Keith, in an address given to the senior nurses of the Rochester (N.Y.) training-schools, reported in the January number of the *American Journal of Nursing*, has good practical advice for caterers of institutions. Meatless days are to be cheerfully observed on the other side, for many entire herds have been converted into beef on account of the scarcity of feed for the cattle, and preserved meats, pork, and bacon are almost as dear as in this country. Perishable articles which cannot be used for the relief of the Allies are to be the housekeeper's main source of supply. Wheat and sugar are to be used very sparingly, since shipping is sternly limited "and wheat packs much nutritive value in small space." Laundry work is to be economised to the fullest extent, and Miss Keith believes that "the amount of clean linen used in our public wards could be cut down one-half without lessening our efficiency." The shortage of labour is already deplorable, and likely to grow worse. Grain has been rotting in the West for lack of harvesters, and peaches were offered for twenty-five cents a bushel to any who would gather and carry them away.

---

The Trained Nurses' Association of South Africa is going through a troubled time in regard to the colour question. It appears there is an enormous demand for native and coloured nurses for work in locations, mine hospitals, etc., there being in the Cape alone 1,800,000 natives to 500,000 whites. The difficulty is that though a very few native women are capable of taking full training and passing an examination for registration, the majority are not yet educated up to the full examination. The proposal has been made that a second-grade native nurse should be recognised to undertake work analogous to that performed by village nurses at home. The many difficulties involved in the recognition of partially trained nurses are fully recognised and are receiving careful attention, but it seems very doubtful whether native women admittedly incapable of receiving a sound training ought ever to be dignified with the title of nurse, even "second-grade." In any case the Trained Nurses' Association is never likely to meet with a more thorny question to handle. It is one which is rising into prominence in many parts of the Empire.

---

Much interest attaches to the Bill to provide for the State Registration of Nurses in the State of Victoria presented by the Minister of Public Health to the Legislative Assembly in August last. The Bill proposes to place the administration of the Act in the hands of a Board consisting of five medical members of the State Public Service, but grave objection is being taken to this proposal even by medical men, and still more, it may be surmised, by the heads of the nurse-training schools in Victoria. News travels slowly through from the Antipodes in these days, but under date of October the news was not very reassuring. The Chief Secretary, Mr. D. M'Leod, in receiving deputations urging, among other points, that nurses should be represented on the proposed Nurses' Board, laid stress on the fact that nurses were to be represented on the Board of Examiners, but adhered to the purely medical composition of the Board of Control. It seems extraordinary from an English point of view that it was left to the doctors and chemists to bring the case for nursing representation on the Board of Control before the Minister of Health.

---

☞ It is our invariable practice to pay for all items of news which we may publish. The minimum payment is 5s. Paragraphs of about twenty lines in length—that is to say, of 150 words or less—are preferred. Contributions should be addressed to The Editor, The Hospital, 28 and 29 Southampton Street, Strand, London, W.C. 2, and, to be in time for the current week's issue, should reach him by the first post on Mondays.

For Matrons' and Sisters' Appointments, see p. 448; "The Shortage of Husbands, see p. 447.

